# Patient perceptions and experiences of dental fear of different dental specialties: a mixed-method study

**DOI:** 10.1186/s12903-023-03626-3

**Published:** 2023-11-19

**Authors:** Muhammad Taqi, Syed Jaffar Abbas Zaidi, Javaria Javaid, Zainab Alam, Aimen Saleem, Sadia Asghar Khan

**Affiliations:** 1 Department of Community Dentistry, Dow Dental College, Dow University of health sciences Karachi, Karachi, Pakistan; 2 Department of Oral Biology, Dow Dental College, Dow University of health sciences Karachi, Karachi, Sindh 74200 Pakistan

**Keywords:** Dental fear, Dental fear survey, Patient interview

## Abstract

**Objective:**

The primary objective of this study was to validate an Urdu translation of Kleinknecht’s Dental Fear Survey (DFS) for use in Pakistan and to explore which items contribute the most to the variance in dental fear scores based on patient perceptions and lived experiences during dental care.

**Methodology:**

This mixed-method study was conducted at Dow Dental Hospital from February 2022 to June 2022. For quantitative analysis, a total of 273 participants were enrolled through convenience sampling. After obtaining signed consent, participants were asked to self-report their dental fear. In-depth interviews with 25 patients displaying moderate to high dental fear were conducted to clarify the elements of dental fear scores through the lens of individual perceptions and experiences.

**Results:**

The prevalence of moderate dental fear was significantly higher among female participants than males. The mean dental fear score was higher among females (39.47 ± 14.23) as compared to males (30.83 ± 10.50). Most of the female participants reported an increase in breathing rate and heartbeat during dental treatment. The highest mean fear score was reported by participants who underwent oral surgical treatment (42.98 ± 14.21), followed by participants who received restorative care (36.20 ± 12.60). Approaching the dentist’s office was the significant factor that contributed the most to the variance in dental fear scores. Four themes were generated through the content analysis of the interviews: physical reactions to dental procedures, perceptions and fears about surgical and restorative procedures, and gender and environmental factors in dental fear and interaction with dentists.

**Conclusion:**

The Urdu translation of DFS is a reliable and valid instrument for assessing dental fears in Pakistan based on the findings of this study. Patients perceive surgical and restorative procedures as unpleasant and threatening. It was noted that “the heart beats faster” and “the breathing rate increases.“ were the top two physiological responses.

## Introduction

Technological advances in health and enhanced understanding of patients’ needs have not eliminated or substantially reduced dental fear and anxiety [1]. The literature reports that dental fear and dental anxiety are two separate psychological states. Dental anxiety is a vague, unpleasant feeling or stress that people experience during a dental appointment that something undesirable will happen [2]. On the other hand, dental fear is a state of human physiological, behavioural, and emotional response to a threatening stimulus such as needles or drills in a dental situation that results in avoidance of dental treatment [3]. In the literature, both terms are used interchangeably in studies, but are not the same [4].

Dental fear can significantly impact a patient’s oral, general, and mental health. Patients with dental fear cause them to avoid, cancel, or postponement of regular dental care, resulting in a decline in oral health which then serves to reinforce fear [5, 6]. This decline in health can lead to a decrease in self-respect and negatively affect their phonetics, chewing efficiency, appearance, and social interaction [7–9]. Due to these significant and widespread effects, it is vital to identify patients with dental fear and treat them appropriately in the dental office.

In Pakistan, most people skip regular dental check-ups due to a lack of understanding of their dental needs, cost, limited availability of health care [10]. Toothache is the most common reason to approach a dentist. Most of the population avoids regular dental visits, leading to the advanced stage of decay, which is usually beyond repair. Therefore, on the first visit to a dentist, patients are exposed to the painful procedure of tooth extraction [11] that can develop dental fear and anxiety. A cross-sectional study on Pakistani population reported that the most common cause of avoiding dental visits is fear of pain and discomfort [12].

Furthermore, in Pakistan, isolated studies reported a prevalence of moderate to severe dental fear and anxiety ranging from 23 to 38% [13, 14]. Although anxiety and fear are conceptually different, the available studies in the Pakistani population reported dental anxiety and fear using single anxiety scales [13–15]. Dental fear has subjective (emotions and cognitions) and objective (behaviour and physiological reactions) components [16]. Kleinknecht’s Dental Fear Survey (DFS) [17] has been widely used to assess dental fear in international epidemiological studies for more than 30 years [18, 19]. Research studies have found that it has high stability, reliability, and acceptable validity in cultures and languages [20].

Understanding patients’ subjective dental fear and the stimuli that trigger it is crucial to determine appropriate treatment plans and fostering positive motivation for future dental visits. So far, there is no evidence of using dental fear screening through the Kleinknecht Dental Fear Survey (DFS) in Pakistan. A lack of research on the varying levels of dental fear associated with different types of dental procedures has also not reported in existing Pakistani dental fear studies. These studies primarily focus on the fear of patients presenting for dental treatment.

Therefore, this study has four primary objectives (1) To validate an Urdu translation of Kleinknecht’s Dental Fear Survey (DFS). (2) To compare the DFS scores among patients undergoing treatments across various dental specialties. (3) To identify the DFS items that account for the most significant variance in the overall DFS scores. (4) To conduct interviews with individuals exhibiting moderate to high dental fear, aiming to gain insights into their perceptions of specific DFS items based on their personal experiences.

## Methods

### Study design and setting

This mixed methods study was conducted in the outpatient departments of five dental specialties (Oral surgery, Restorative dentistry, Periodontology, Prosthodontics, Orthodontics) of Dow Dental College from February 2022 to June 2022, and participants were offered no compensation. The first part of the study was a quantitative descriptive cross-sectional study. Participants were enrolled through convenience sampling. The total number of participants required for this study was 273. The sample size was calculated using the reported prevalence of dental fear of 23% [13] based on a margin of error of 5% and a 95% confidence interval (CI). Later the 5 strata for five dental specialities were formed each contain 54 participants.

The participants aged 18 years or above who provided consent and could complete questionnaires independently were included. In contrast, the investigators excluded the participants with physical and mental disabilities, patients with psychiatric illnesses, terminally ill patients, patients with alcohol or drug dependency, and patients receiving dental care under general anaesthesia. The physical and mental disabilities were confirmed from the patients history forms. The questionnaire was administered when participants were seated on a dental chair before the clinical dental examination, and study information was provided in written and verbal form. After obtaining signed consent, participants were asked to self-report their dental fear, answering a given questionnaire. Upon providing their consent, participants were apprised of the possibility of being invited for a subsequent interview.

The second part of the study was qualitative in-depth face to face interviews based on the study results. Twenty-five patients were selected who had moderate to high dental fear scores. Interviews were conducted in person subsequent to the calculation of dental fear scores and following the participants’ initial dental visits, but prior to their subsequent dental appointments. The items that contributed the most to the variance in dental fear scores were investigated through in-depth face to face patient interviews. Participants were asked to share their perspectives and rich experiences of dental fear through semi-structured interviews. A single investigator conducted each interview, which lasted 45 min to an hour.

### Study instrument

The questionnaire consisted of three parts. The first section explains the project’s objectives, the process of answering the questions, and the consent form. In section two, respondents were asked for demographic information. Section three contains 20 questions related to dental fear. The dental fear questions were divided into three domains (1) avoidance of dental visits (8 questions), (2) physiological reactions (5 questions), and (3) fear of specific dental stimuli (7 questions).

In section three, questions with five-point Likert scale ranging from 1 to 5 (1: never, 2: once or twice, 3: a few times, 4: often, 5: nearly every time) was used to assess the reasons for avoiding dental visit and physiological reactions. Furthermore, a five-point Likert scale ranging from 1 to 5 (1: very relaxed, 2: low fear, 3: afraid, 4: very afraid, 5: Being so anxious I feel ill) was used to assess the dental fear from a specific stimulus [21]. Avoidance scores ranged from 8 to 40, physiological arousal from 5 to 25, and fears of specific stimuli/situations from 7 to 35 [19]. The dental fear survey (DFS) score ranged from 0 to 20 (no fear), 21–40 (low fear), 41–60 (moderate fear), 61–80 (high fear), and > 80 (extreme fear) [22, 23].

### Questionnaire validation

The original Dental Fear Survey by Kleinknecht [17] was translated into Urdu. Two bilingual translators, native to the Urdu language, were involved: One informed translator (a dentist) familiar with dental terminology. One uninformed translator (non-medical but expert in both English and Urdu languages). An expert health professional, not involved in the translation process, evaluated the content validity of the translated version. This step ensured that the questionnaire adequately represented the construct it aimed to measure.

The Urdu-translated questionnaire was pretested on 25 participants not part of the main study.

Objectives of the pre-test included assessing: Comprehension by participants and time taken for completion. On average, participants took six minutes to fill out the form.

Internal consistency was determined using Cronbach’s alpha. This assessment gauged the consistency between responses to the complete DFS and responses to the questions across all three domains (refer to Table 1).

The Pearson’s correlation coefficient (𝑟) was computed between individual items and the aggregate scores of respondents (as detailed in Table 2). Items with correlation values exceeding the threshold of 0.39 were considered to demonstrate strong validity. This threshold was derived from the critical values table for Pearson correlation, which is instrumental in determining statistical significance. After analysing the pilot data and obtaining feedback, the final version of the Urdu-translated Dental Fear Survey was drafted, ready for the main study.

### Data analysis

#### Quantitative analysis

Quantitative data analysis was conducted using IBM SPSS statistics version 21. The chi-square test was used to assess the frequency distribution of participants according to gender in each dental speciality (Table 3). A similar test was used to estimate the frequency distribution of participant responses to situations, feelings, and responses to dental work (Table 4) and the distribution of participants according to the rate of fear caused by the situations faced in the dental office (Table 5). An independent-sample t-test was used to compare mean scores of avoidances, physiological arousal, and fear domains between genders and dental specialities. Multiple regression analysis determined the variable contributing the most variance in a dental fear score. A level of significance was established at less than 0.05.

#### Qualitative analysis

**Fig. 1:**
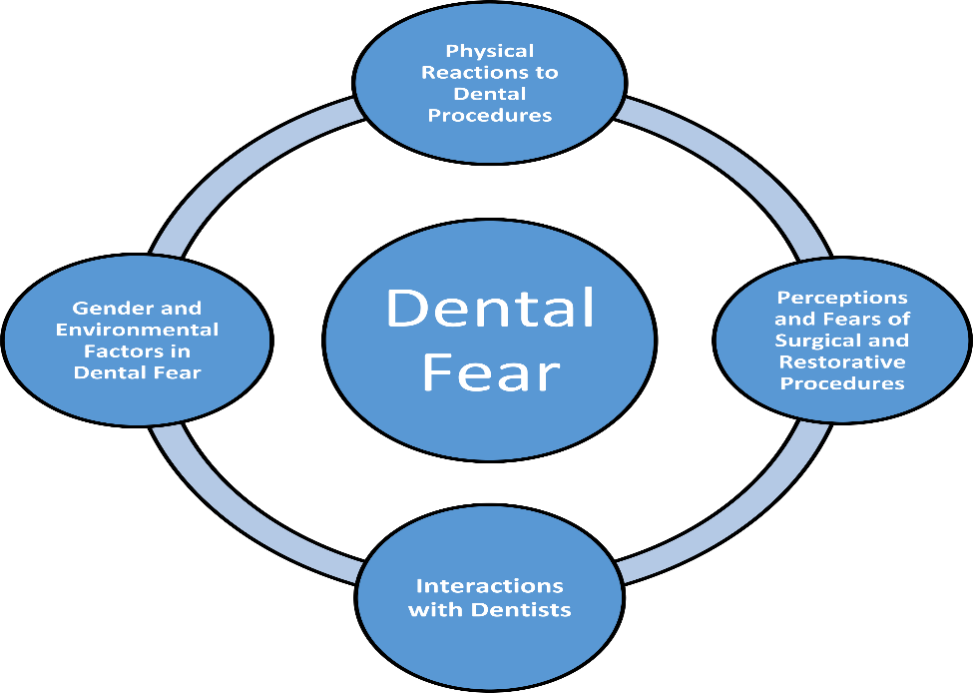
Themes of dental fear identified from individuals with moderate to high dental fear

The interview analysis was conducted concurrently with the inductive interpretation of the data. The principal investigator recorded all the interviews using Audacity software to remove the pauses and improve the sound quality. The files were then converted to mp3 versions and then transcribed accordingly. Then, a manual analysis of the interviews was conducted. Themes and codes were generated from the transcribed interviews, as shown in Table 9. After identifying the categories, representative sentences were highlighted in the transcripts. All authors interpreted and coded the data, and themes were agreed upon through discussion. During the coding process, data were accumulated, and thematic analysis was conducted. Constant comparative analysis was used to continuously compare the themes with the data based on grounded theory principles. Disconfirmatory evidence was sought in subsequent interviews after thematic categories in the initial interviews. The data of each group supported a set of master themes as shown in Table 9; Fig. 1.

The sample size for the interviews was determined through thematic saturation, a process in which new data appear to no longer contribute to the findings due to the repetition of themes and comments from participants. The data generation process was terminated at this point. A total of 25 patients were included in the final sample. Interviews were completed with 25 patients; however, data saturation in qualitative terms was reached with the first 20 patients. No additional categories or themes emerged from the subsequent five interviews. Consequently, the thematic analysis focused on the pre-established codes derived from the initial 20 participants. Quantitative and qualitative data from were then integrated and analysed accordingly.

## Results

### Validation of the urdu translation of Kleinknecht’s dental fear survey

Table 1 presents the reliability analysis of the translated version of the Dental Fear Survey (DFS) using Cronbach’s alpha (α) coefficient. The complete DFS, which consists of 20 questions, demonstrates a good internal consistency with a Cronbach’s alpha of 0.82. The mean score for the complete survey is 43.39 with a standard deviation of 11.38.

When the DFS is broken down into its subcomponents, each section also exhibits satisfactory reliability. The “Avoidance” section, comprising 8 questions, has a Cronbach’s alpha of 0.70 and a mean score of 14.85 (± 5.17). The “Physiological arousal” section, containing 5 questions, has a slightly higher Cronbach’s alpha of 0.71, with participants scoring an average of 9.08 (± 3.86). Lastly, the “Fears of specific stimuli/situations” section, which consists of 7 questions, demonstrates a Cronbach’s alpha of 0.79. The mean score for this section is 19.46 with a standard deviation of 5.76.

The translated version of the DFS appears to maintain good reliability across its complete set of questions and its subsections. This suggests that the translated version is a consistent tool for measuring dental fear in the sample population.

**Table 1:** Cronbach’s alpha (α) coefficient for the translated version of the dental fear survey (DFS)(n = 25)

	**Questions**	**Cronbach’s Alpha**	**Mean ± S.D**
Complete DFS	20	0.82	43.39 ± 11.38
Avoidance	8	0.70	14.85 ± 5.17
Physiological arousal	5	0.71	9.08 ± 3.86
Fears of specific stimuli/situations	7	0.79	19.46 ± 5.76

Table 2 details the Pearson correlation coefficients for questions spanning the three domains of the Dental Fear Survey: Avoidance, Physiological arousal, and Fears of specific stimuli/situations.

In the “Avoidance” domain, all questions showed significant positive correlations with dental fear, with the obtained values ranging from 0.407 to 0.729. Notably, questions relating to “Approaching the dentist’s office” and “Seeing the dentist walk-in” exhibited particularly strong correlations, with coefficients of 0.638 and 0.729, respectively, both with p-values of 0.001.

Within the “Physiological arousal” domain, all questions again demonstrated significant positive correlations with dental fear. The strongest correlation was observed for the statement “I feel nauseated and sick to my stomach,“ with a correlation coefficient of 0.790 (p = .001).

The “Fears of specific stimuli/situations” domain also presented substantial positive correlations across all questions. Particularly potent correlations were observed for “Seeing the drill” and “Feeling the vibrations of the drill,“ with coefficients of 0.773 and 0.577, respectively, both with p-values indicating high significance.

It’s worth noting that all obtained values in the table exceeded the critical value of 0.39, signifying meaningful correlations. The p-values associated with each correlation coefficient further validate the significance of these correlations, with most of them being less than 0.05 and a majority even less than 0.01.

The questions across all three domains of the Dental Fear Survey exhibit significant positive correlations with dental fear, indicating that these questions are effective indicators of dental fear and anxiety in the studied population.

**Table 2:** Pearson correlation coefficients for questions in all three domains using a degree of freedom two and critical value (0.39)

Avoidance	Mean ± S.D	Obtained value	*p*-value
Has fear of dental work ever caused you to put off making an appointment?	1.72 ± 1.17	0.509	0.009
Has fear of dental work ever caused you to cancel or not appear for an appointment?	1.52 ± 1.15	0.452	0.023
Making an appointment for dentistry	2.32 ± 1.14	0.457	0.022
Approaching the dentist’s office	1.76 ± 1.24	0.638	0.001
Sitting in the waiting room	2.00 ± 1.08	0.490	0.013
Being seated in the dental chair	1.96 ± 1.24	0.724	0.001
The smell of the dentist’s office	1.80 ± 1.04	0.407	0.044
Seeing the dentist walk-in	1.77 ± 1.21	0.729	0.001
**Physiological arousal. When having dental work done**			
My muscles become tense	1.80 ± 1.04	0.484	0.014
My breathing rate increases	1.88 ± 1.23	0.781	0.001
I perspire	1.64 ± 1.08	0.705	0.001
I feel nauseated and sick to my stomach	1.92 ± 1.15	0.790	0.001
My heart beats faster	1.84 ± 1.10	0.648	0.001
**Fears of specific stimuli/situations**			
Seeing the anaesthetic needle	3.39 ± 1.13	0.641	0.001
Feeling the needle injected	3.42 ± 1.09	0.663	0.001
Seeing the drill	2.83 ± 1.40	0.773	0.001
Hearing the drill	2.79 ± 1.33	0.724	0.001
Feeling the vibrations of the drill	2.43 ± 1.07	0.577	0.003
Having your teeth cleaned	2.05 ± 1.20	0.641	0.001
All things considered, how fearful are you of having dental work done?	2.56 ± 1.37	0.634	0.001

#### Comparison of DFS scores across dental specialties

Out of 300 participants, 273 completed the survey with a response rate of 91%, remaining 27 participants did not submit the response. Out of 273, male participants were 134(49%), and female participants were 139(50.9%). The overall mean fear score was 35.23 ± 13.24. The mean dental fear score was higher among females (39.47 ± 14.23) as compared to males (30.83 ± 10.50). The mean score reported by participants who received dental treatment in periodontology was 30.06 ± 10.44, oral surgery was 42.98 ± 14.21, restorative was 36.20 ± 12.60, prosthodontics 34.40 ± 14.95 and orthodontics 32.44 ± 9.92.

The prevalence of dental fear was significantly higher among female participants, 94.9%, compared to male participants, 86.5% (p = .001). A significant number of female participants reported moderate levels of fear before receiving surgical treatment 20(80%), *p* = .001(Table 3).

Most female participants reported increased breathing rate and heartbeat during dental treatment (Table 4). The data revealed variations in the frequency of fear-related behaviors across different scenarios. When considering the impact of dental fear on appointment scheduling, it was found that a higher proportion of males reported delaying appointments due to fear, although this difference was not statistically significant (p = .088). Similarly, a larger percentage of males indicated cancelling or not appearing for appointments due to fear, though the difference was not statistically significant (p = .206).

**Table 3:** Gender-wise distribution of participants enrolled from five dental specialities and level of fear

Dental speciality	Male N(%)	Female N(%)	p-value
Periodontology No Fear	5 (15.1)	4(18.1)	0.510
Low Fear	24(72.7)	12(59)	
Moderate Fear	4(12.1)	5(22.7)	
High Fear	0	0	
Total	33(100)	21(100)	
Oral Surgery No Fear	1(3.3)	0	0.001
Low Fear	20(66.6)	4(20)	
Moderate Fear	8(26.6)	20(80)	
High Fear	1(3.3)	0	
Total	30(100)	24(100)	
Restorative No Fear	3(11.1)	1(3.5)	0.001
Low Fear	22(81.4)	11(42.8)	
Moderate Fear	2(7.4)	15(53.5)	
High Fear	0	0	
Total	27(100)	27(100)	
Prosthodontics No Fear	6(13)	1(3.2)	0.019
Low Fear	14(60)	19(61.2)	
Moderate Fear	3(13)	11(35.4)	
High Fear	0	0	
Total	23(100)	31(100)	
Orthodontics No Fear	2(9.5)	1(3)	0.274
Low Fear	17(80.9)	24(72.7)	
Moderate Fear	2(9.5)	8(24.2)	
High Fear	0	0	
Total	21(100)	33(100)	
Overall No Fear	17(12.7)	7(5)	0.001
Low Fear	97(72.4)	73(52.5)	
Moderate Fear	19(14.2)	59(42.4)	
High Fear	1(0.7)	0	
Total	134(100)	139(100)	
Chi-square Test			

**Table 4:** Participants’ responses to the situation, feelings and responses to dental work

	Gender N(%)	Never N(%)	Once / Twice N(%)	Few times N(%)	Often N(%)	Nearly every time N(%)	P value
1. Has a fear of dental work ever caused you to put off making an appointment?	Male	102(76.1)	14(10.4)	13(9.7)	2(1.5)	3(2.2)	0.088
	Female	93(66.9)	16(11.5)	13(99.4)	12(8.6)	5(3.6)	
2. Has a fear of dental work ever caused you to cancel or not appear for an appointment?	Male	106(79.1)	17(12.7)	3(2.2)	6(4.5)	2(1.5)	0.206
	Female	96(69.1)	22(15.8)	11(7.9)	8(5.8)	2(1.4)	
*When having dental work done: 3. My muscles become tense	Male	102(76.1)	20(14.9)	7(5.2)	4(3.0)	1(0.7)	0.068
	Female	92(66.2)	17(12.2)	20(14.4)	7(5.0)	3 (2.2)	
4. My breathing rate increases	Male	105(78.4)	18(13.4)	4(3.0)	3(2.2)	4(3.0)	0.018
	Female	84(60.4)	29(20.9)	8(5.8)	12(8.6)	6(4.3)	
5. I perspire	Male	119(88.8)	6(4.5)	5(3.7)	3(2.2)	1(0.7)	0.053
	Female	107 (77)	11(7.9)	5(3.6)	10(7.2)	6(4.3)	
6. I feel nauseated and sick to my stomach.	Male	115 (85.8)	11(8.2)	0(0)	7(5.2)	1(0.7)	0.060
	Female	105(75.5)	17(12.2)	7(5)	8(5.8)	2(1.4)	
7. My heartbeats faster	Male	92(68.7)	23(17.2)	9(6.7)	9(6.7)	1(0.7)	0.022
	Female	74(53.2)	26(18.7)	14(10.1)	16(11.5)	9(6.5)	
Chi-square Test							

Most participants reported a low level of fear in a fear-producing situation (Table 5). The data indicates differing levels of fear and anxiety experienced in these scenarios. For instance, in situations such as making an appointment, approaching the dentist’s office, and being seated in the waiting room, more females expressed higher fear levels compared to males, with statistically significant differences (p < .05). Similar trends were observed when considering reactions to stimuli like the smell of the dentist’s office, seeing the dentist, the anaesthetic needle, and various aspects of dental procedures. In general, the findings highlight gender-related variations in fear responses to specific dental situations, emphasizing potential psychological and emotional differences among male and female participants.

**Table 5:** Participants’ responses to a list of fear-producing situations

	Gender N(%)	Very relaxed N(%)	Low fear N(%)	Afraid N(%)	Very afraid N(%)	Being so anxious, I feel ill N(%)	*p-*value
8. Making an appointment for dentistry	Male	81(60.4)	45(33.6)	6(4.5)	1(0.7)	1(0.7)	0.003
	Female	58(41.7)	53(38.1)	18(12.9)	7(5)	3(2.2)	
9. Approaching the dentist’s office	Male	93(69.4)	30(22.4)	6(4.5)	4(3)	1(0.7)	0.003
	Female	64(46)	49(35.3)	16(11.5)	8 (5.8)	2(1.4)	
10. Sitting in the waiting room.	Male	100(74.6)	26(19.4)	5(3.7)	2(1.5)	1(0.7)	0.025
	Female	82(59)	38(27.3)	6(4.3)	12(8.6)	1(0.7)	
11. Being seated in a dental chair	Male	85(63.4)	40(29.9)	6 (4.5)	2 (1.5)	1 (0.7)	0.007
	Female	59(42.4)	56 (40.3)	16(11.5)	6 (4.3)	2 (1.4)	
12. The smell of the dentist’s office.	Male	104(77.6)	20(14.9)	8(6)	1(0.7)	1(0.7)	0.017
	Female	83(59.7)	31(22.3)	15(10.8)	5(3.6)	5(3.6)	
13. Seeing the dentist walk in.	Male	109(81.3)	17 (12.7)	5 (3.7)	3 (2.2)	0 (0)	0.002
	Female	82 (59)	38 (27.3)	11 (7.9)	6 (4.3)	2 (1.4)	
14 Seeing the anaesthetic needle	Male	45(33.6)	49(36.6)	27(20.1)	5(3.7)	8(6)	0.001
	Female	20(14.4)	45(32.4)	31(22.3)	18(12.9)	25(18)	
15 Feeling the needle injected	Male	40(29.9)	49(36.6)	24(17.9)	14(10.4)	7(5.2)	0.001
	Female	18(12.9)	51(36.7)	28(20.1)	16(11.5)	26(18.7)	
16 Seeing the drill	Male	61(45.5)	45(33.6)	13(9.7)	13(9.7)	2(1.5)	0.001
	Female	33(23.7)	45(32.4)	32(23)	15(10.8)	14(10.1)	
17 Hearing the drill	Male	70(52.2)	36(26.9)	21(15.7)	6(4.5)	1(0.7)	0.001
	Female	41(29.5)	44(31.7)	29(20.9)	13(9.4)	12(8.6)	
18. Feeling the vibrations of the drill	Male	60(44.8)	48(35.8)	15(11.2)	10(7.5)	1(0.7)	0.001
	Female	30(21.6)	51(36.7)	29(20.9)	15(10.8)	14(10.1)	
19. Having your teeth cleaned.	Male	91(67.9)	25(18.7)	12(9)	5(3.7)	1(0.7)	0.001
	Female	56(40.3)	47(33.8)	22(15.8)	6(4.3)	8(5.8)	
20. All things considered, how fearful are you of having dental work done?	Male	70(52.2)	51(38.1)	9(6.7)	3(2.2)	1(0.7)	0.001
	Female	39(28.1)	60(43.2)	18(12.9)	14(10.1)	8(5.8)	
Chi-square Test							

Table 6 presents a gender-wise comparison of mean scores in three distinct domains related to dental fear: Avoidance, Physiological Arousal, and Fear of Specific Stimuli. The data reveals significant differences in mean scores between males and females across all three domains, with p-values less than 0.001. Specifically, females tend to report higher mean scores in all domains compared to males. These findings suggest that females exhibit higher levels of dental fear in each of the measured domains, underscoring potential gender-related differences in dental fear experiences.

**Table 6:** Gender-wise comparison of mean scores in three domains

Domain	Avoidance		Physiological arousal		Fear of specific stimuli	
Gender	Mean ± S.D	*p*-value	Mean ± S.D	*p*-value	Mean ± S.D	*p*-value
Male	11.1 ± 4.03	0.001	12.9 ± 5.20	0.001	6.77 ± 2.93	0.001
Female	13.7 ± 5.54		17.3 ± 7.00		8.38 ± 3.79	
Overall	12.43 ± 5.02		15.19 ± 6.55		7.59 ± 3.49	
Independent Sample *t* test						

Table 7 provides a dental speciality-wise comparison of mean fear scores within three distinct domains: Avoidance, Physiological Arousal, and Fear of Specific Stimuli. The data reveals variability in mean fear scores across different dental specialities. In the Avoidance domain, participants in the Periodontology speciality had the lowest mean fear score (10.85 ± 3.71), significantly differing from participants in the Oral Surgery (p = .001), Restorative (p = .009), and Prosthodontics (p = .085) specialities. A similar trend is observed in the Physiological Arousal domain, with Periodontology having the lowest mean fear score (6.32 ± 2.24) and statistically significant differences compared to other specialities. In the Fear of Specific Stimuli domain, Periodontology also had the lowest mean fear score (12.83 ± 5.66) and significant differences with Oral Surgery (p = .001), Restorative (p = .028), and Prosthodontics (p = .210). These findings suggest that participants from the Periodontology speciality tend to exhibit comparatively lower levels of fear across all three domains, potentially reflecting variations in dental experiences and expectations among different dental specialities.

**Table 7:** Dental speciality-wise comparison of mean fear scores in three domains

Domain Avoidance		Periodontology	Oral Surgery	Restorative	Prosthodontics	Orthodontics
	Mean ± S.D	10.85 ± 3.71	14.72 ± 5.78	13.01 ± 4.76	12.42 ± 5.56	11.12 ± 4.16
Periodontology	10.85 ± 3.71	-	0.001	0.009	0.085	0.716
Oral Surgery	14.72 ± 5.78	0.001	-	0.094	0.037	0.001
Restorative	13.01 ± 4.76	0.009	0.094	-	0.552	0.030
Prosthodontics	12.42 ± 5.56	0.085	0.037	0.552	-	0.173
Orthodontics	11.12 ± 4.16	0.716	0.001	0.030	0.173	-
Domain Physiological Arousal		Periodontology	Oral Surgery	Restorative	Prosthodontics	Orthodontics
	Mean ± S.D	6.32 ± 2.24	9.30 ± 4.48	7.74 ± 2.93	7.64 ± 4.07	6.94 ± 2.51
Periodontology	6.32 ± 2.24	-	0.001	0.005	0.039	0.179
Oral Surgery	9.30 ± 4.48	0.001	-	0.033	0.045	0.001
Restorative	7.74 ± 2.93	0.005	0.033	-	0.883	0.129
Prosthodontics	7.64 ± 4.07	0.039	0.045	0.883	-	0.285
Orthodontics	6.94 ± 2.51	0.179	0.001	0.129	0.285	-
Domain Fear of specific stimuli		Periodontology	Oral Surgery	Restorative	Prosthodontics	Orthodontics
	Mean ± S.D	12.83 ± 5.66	18.94 ± 6.99	15.45 ± 6.59	14.33 ± 6.69	14.37 ± 5.21
Periodontology	12.83 ± 5.66	-	0.001	0.028	0.210	0.144
Oral Surgery	18.94 ± 6.99	0.001	-	0.008	0.001	0.001
Restorative	15.45 ± 6.59	0.028	0.008	-	0.380	0.344
Prosthodontics	14.33 ± 6.69	0.210	0.001	0.380	-	0.974
Orthodontics	14.37 ± 5.21	0.144	0.001	0.344	0.974	-

#### Identifying key DFS items influencing overall scores

According to the stepwise multivariate regression analysis results, approaching the dental office has a significant effect size (beta coefficient) on the development of dental fear (Table 8). These findings suggests that individuals who reported certain perceptions or experiences related to approaching the dental office also tended to exhibit higher levels of dental fear, according to the analysis. However, due to the cross-sectional nature of this study, it is important to note that the observed association does not imply a causal relationship between approaching the dental office and the development of dental fear. While this finding provides valuable insights into the potential relationship, further research, such as longitudinal studies or experimental designs, is needed to explore the temporal and causal aspects of this association in greater detail.“

**Table 8:** Items in a forward stepwise regression mode

Variable	t	Standardise Beta	*p*-value	*F*
Seeing the drill	5.70	0.161	0.001	395.3
Approaching the dentist’s office	7.90	0.175	0.001	560.7
Being seated in the dental chair	4.89	0.104	0.001	521.0
Feeling the needle injected	4.86	0.122	0.001	496.3
Have your teeth cleaned	5.68	0.107	0.001	477.5
The smell of the dentist’s office	4.48	0.087	0.001	454.1
Making an appointment	5.68	0.120	0.001	441.2
Feeling the vibration of the drill	4.36	0.121	0.001	428.5
Seeing the dentist walk-in	4.29	0.081	0.001	416.0
Seeing the anaesthetic needle	3.90	0.096	0.001	398.1
Sitting in the waiting room	3.91	0.082	0.001	382.3
Hearing the drill	2.55	0.076	0.011	358.5

Twenty patients with high dental fear scores were selected, and their perceptions and lived experiences were then explored through in-depth interviews, as shown in Table 9. The items that contributed the most to the variance in dental fear scores were investigated through these interviews. Four themes were generated from the qualitative content analysis as shown in Fig. 1. These themes were physical reactions to dental procedures, perceptions and fears of surgical and restorative procedures, and gender and environmental factors in dental fear and interaction with dentists.

**Table 9:** Interview insights on DFS items from individuals with moderate to high dental fear

Themes	Categories	Codes	Representative Sentences
Physical Reactions to Dental Procedures	Increased Heart Rate	DS 1.14	“I can feel my heart pounding in my chest as soon as I sit in the dentist’s chair.“
	Increased Breathing Rate	OP 1.20	“My breathing becomes erratic and shallow when I’m undergoing a dental procedure.“
Perceptions and Fears of Surgical and Restorative Procedures	Existential Threat	DS 2.16	“I’m always scared that something might go terribly wrong during the procedure.“
	Unpleasantness of Procedures	OP 2.06	“The noise of the drill, the smell of the clinic, everything is just so unpleasant.“
Gender and Environmental Factors in Dental Fear	Female Admission of Fear	DS 3.09	“As a woman, I’m not ashamed to admit that I’m scared of dental procedures.“
	Male Reluctance to Admit Fear	OP 3.04	“I don’t really like admitting it, but I am terrified of the dentist.“
	Approach-related Fear	DS 3.18	“The mere thought of approaching the dentist’s office makes me nervous.“
Interactions with Dentists	Perceived Lack of Empathy	DS 4.19	“I feel like the dentist doesn’t really care about my fear or try to comfort me.“
	Vulnerability in the Dental Chair	OP 4.07	“Sitting in the dental chair, I feel exposed and helpless, which just increases my fear.“

### Physical reactions to dental procedures

Two categories were identified in the physical reactions to dental procedures. These were the increased heart and breathing rates that the patients experienced during dental care. These physiological conditions can be attributed to fear, apprehension, and anxiety associated with dental procedures. Some patients even reported feeling of impending doom when they sat in the dental chair.

These findings highlight the need for dentists to understand dental fear before starting any dental procedure, as stress can lead to life-threatening emergencies in the dental office.

“I can feel my heart pounding in my chest as soon as I sit in the dentist’s chair.“ (DS 1.14).

“My breathing becomes erratic and shallow when I’m undergoing a dental procedure.“ (OP 1.20).

### Perceptions and fears of surgical and restorative procedures

The two categories under this theme were existential threat and procedures’ unpleasantness. Existential threat refers to the fear of serious harm or even death stemming from a dental procedure. Existential threats can be triggered by past traumas, hearing of negative experiences from others, being misinformed about dental procedures, or having phobic reactions. The threat is often rooted in a feeling of lack of control and vulnerability when seated in the dental chair. This vulnerability, combined with the proximity of the dentist during procedures and the invasive nature of dental work, can create feelings of existential threat. It can also be exacerbated by the perception of dental procedures as life-threatening, even if this perception is inaccurate or based on outdated information on dental safety.

“I’m always scared that something might go terribly wrong during the procedure.“ (DS 2.16).

The unpleasantness of dental procedures revolves around the discomfort and pain associated with dental treatments. Many people fear the pain of injections or the discomfort of having someone work in their mouth. This fear might be triggered by past painful experiences, the sight, sound, or sensation of the dental drill, or the mere anticipation of pain, sometimes worse than the actual sensation of pain itself. Moreover, the fear of potential side effects such as numbness, difficulty speaking or eating, or drooling due to local anaesthesia can further increase the perception of the unpleasantness of dental procedures.

“The noise of the drill, the smell of the clinic, everything is just so unpleasant.“ (OP 2.06).

### Gender and environmental factors in dental fear

Three categories were identified under this theme: female admission of fear, male reluctance to admit fear and approach-related fear.

In this study, women were generally more open and willing to admit their fears and anxieties related to dental procedures. Their dental fears are usually addressed with ease by healthcare providers, seeking emotional support and actively seeking reassurance.

“As a woman, I’m not ashamed to admit that I’m scared of dental procedures.“ (DS 3.09).

Men, on the other hand, often exhibit a greater reluctance to admit their fear or anxiety surrounding dental procedures. Societal expectations of stoicism and the pressure to appear tough can lead them to downplay or suppress their dental anxieties, hindering their ability to seek support and address their concerns effectively.

“I don’t really like admitting it, but I am terrified of the dentist.“ (OP 3.04).

Approach-related fear refers to the specific fear or anxiety individuals experience when faced with the prospect of approaching a dentist or dental environment. It can stem from past traumatic experiences, anticipated discomfort, fear of injections or dental instruments, and a perceived loss of control. Addressing this fear requires effective communication, patient education, and implementation of anxiety-reducing techniques in dental practices.

“The mere thought of approaching the dentist’s office makes me nervous.“ (DS 3.18).

### Interaction with dentists

The two categories identified under the overarching theme of interaction with dentists are perceived lack of empathy and vulnerability in the dental chair. Feeling vulnerable while sitting in the dental chair may compound these fears and anxieties, leading to avoidance behaviour and poorer oral health. The vulnerability in the dental chair is also a significant issue. The very nature of dental treatment—lying prone in a chair while another person performs procedures in one’s mouth—can inherently feel invasive and uncomfortable. This vulnerability can be amplified if the dentist appears rushed, dismissive, or insensitive to the patient’s comfort and emotional state.

“Sitting in the dental chair, I feel exposed and helpless, which just increases my fear.“ (OP 4.07).

Perceived lack of empathy from dentists can intensify feelings of vulnerability and discomfort. If patients feel their concerns, fears, or physical discomfort are not acknowledged or addressed, it can lead to a breakdown in communication and trust. Empathy in healthcare settings, including dentistry, involves understanding and responding to the patient’s experience, emotions, and perspective. If a patient feels that their dentist lacks empathy, it can increase their fear and make them less likely to seek care.

“I feel like the dentist doesn’t really care about my fear or try to comfort me.“ (DS 4.19).

Therefore, promoting empathic communication and managing patient vulnerability should be integral components of dental care.

## Discussion

To date, there are no reports of dental fear screening in Pakistan using Kleinknecht’s Dental Fear Survey (DFS). Furthermore, studies on dental fear in Pakistan have not delved into the varying levels of dental fear associated with different types of dental procedures. Addressing this gap, the current study set forth with specific objectives: (1) To validate an Urdu translation of Kleinknecht’s Dental Fear Survey (DFS), ensuring its relevance and reliability for the local population; (2) To compare the DFS scores among patients undergoing treatments across various dental specialties, offering insights into potential variations based on the type of treatment; (3) To identify the DFS items that account for the most significant variance in the overall DFS scores, providing clarity on the most influential factors; and (4) To conduct interviews with individuals exhibiting moderate to high dental fear, aiming to gain a deeper understanding of their perceptions of specific DFS items based on their personal experiences. Through these objectives, we aimed to provide a comprehensive perspective on dental fear in the Pakistani context using the DFS.

The primary objective of this study was to validate an Urdu translation of Kleinknecht’s Dental Fear Survey (DFS) for use in Pakistan, a region where no prior assessments using the DFS had been reported. Our findings confirm that the Urdu version of the DFS is both reliable and valid, making it a valuable tool for assessing dental fear in Pakistani populations.

The translated version of the DFS demonstrated good internal consistency, as indicated by a Cronbach’s alpha of 0.82 for the complete survey. This is consistent with the original DFS and other translated versions used in various cultures, suggesting that the Urdu DFS is as reliable as its counterparts. The subsections of the survey also maintained satisfactory reliability, further confirming the consistency of responses across different aspects of dental fear [19].

In terms of construct validity, the strong positive correlations across questions in all three domains of the DFS indicate that the translated items are effectively capturing the essence of dental fear as intended. The correlation values exceeding the critical value of 0.39 further emphasize the meaningfulness of these correlations. This is consistent with the original DFS and other translated versions used in various cultures, suggesting that the Urdu DFS is as valid as its counterparts [19]. Such findings are crucial because they underscore the survey’s ability to distinguish between individuals with varying degrees of dental fear based on their responses.

Comparatively, studies conducted in other regions using the DFS have shown similar patterns of correlations, suggesting that dental fears, regardless of cultural or linguistic differences, have common underlying factors [24–26]. However, it’s essential to acknowledge that certain fears might be more pronounced in specific cultures due to unique cultural or societal influences.

In this study, female participants reported significantly higher mean fear scores in all three domains compared to their male counterparts. This aligns with previous research suggesting that women are more likely to experience dental fear [27–30]. However, the reasons for these gender differences in dental fear are unclear. Liddell and Locker [31] proposed that differences in attitudes towards pain and control among men and women could explain this trend. Furthermore, studies by Eli et al. [32]and Locker et al. [33] found that women tend to recall more pain after dental treatment than men. Manoela TS also identified a more excellent perception of pain stimulation as a confounding factor in women [34]. These findings may be attributed to social stereotypes about gender role behaviour that persist today. However, it is important to note that these results cannot be generalised as Rowe and Moore [35] reported contradictory findings in their study, suggesting that men may be more afraid of dental treatment than women.

The mean scores of the respondents who received oral surgical care were significantly higher in avoidance, physiological arousal, and fear of specific stimuli. The study was conducted in a public dental setting, where departments have long waiting lists, high patient loads, and burnt-out practitioners, leading to adverse patient experiences. Therefore, seeing the dentist walk in and approach the dental clinic can remind patients of past negative experiences that could result in avoiding dental care. Evidence shows that occupational stress among physicians could adversely affect the patients’ care-seeking attitude [36–38].

In the literature, several cut-off points were used to categorise subjects with no, low, and high dental fears to determine the level and prevalence of dental fear. A high cut-off point will lead to a low prevalence, while a low cut-off point will increase prevalence. For ease of comparison, previous studies [3, 17, 18] were used in this study to classify respondents’ levels of fear. The fear level was categorised as 20 (no fear), 21–40 (low fear), 41–79 (moderate fear), 80, and above (high fear). However, some other studies have a different classification of dental fear based on their mean fear scores [2]. Hence, it is essential to be cautious when comparing the results of this study with other studies.

The practice of avoiding or delaying dental treatment seems to be widespread worldwide. Notably, the respondents in this study cancelled their appointments at a lower rate, similar to Japanese or Malaysian populations [2, 39]. The cultural similarities between Asian countries can explain this finding. Like other Asian countries, Pakistanis have great respect for people in authority. Their commitment to honouring appointments is strong once they have been made [2, 40].

Furthermore, our study is conducted at a public sector hospital where most patients are of low socioeconomic status and cannot afford to pay at private clinics. Therefore, the lack of affordability, pain, and discomfort prevent appointment cancellation. Similarly, evidence shows that low-socioeconomic-status families visit a dentist more frequently due to pain or discomfort [10, 41]. Despite this, dental fear is strongly associated with avoidance of dental care [42, 43].

The study population identified “breathing rate increase” and “heartbeat faster” as the most common physiological responses. Several studies have also rated “heartbeat faster” as the most prominent [40, 44, 45]. However, few studies report muscle tenseness as the most frequently reported symptom, followed by “heartbeats faster” [2]. One possible explanation for this phenomenon is that different populations and cultures may respond differently to physiological reactions, since various social groups and cultures accept and interpret dental treatment differently. However, some general trends can be observed regardless of culture or social norms.

Patients undergoing surgical and restorative procedures may experience fear due to the physical arousal caused by pre-operative surgical instruments and set-up, which can serve as a specific stimulus for fear [28]. Dental fear produces a physiological response such as an increase in heart rate (HR) and respiratory rate, as well as an increase in blood pressure, which could cause a hypertensive crisis in a hypertensive patient. Similarly, a relationship has been found between dental fear, diastolic blood pressure, and heart rate [46].

In terms of fear of specific fear stimuli, surgical motors, handpieces, drills, dental syringes, and needles mostly provoke fear, and lead to discomfort in patients [47]. A substantial body of evidence suggests that seeing needles and drills arouse pain and raise fear of unclean or unsterilised instruments [48]. Furthermore, for some patients, visiting the dentist could lead to fear that dentists unnecessarily pull teeth, leading them to avoid dental care.

In this study, patients who received periodontal, orthodontic, and prosthodontic care reported lower mean scores in three domains than those who received restorative and surgical care. Numerous studies have demonstrated reduced levels of dental fear among patients undergoing noninvasive dental treatments (not requiring local anaesthesia and drills) [48]. Patients receiving treatments from departments that typically provide noninvasive procedures (that do not require local anaesthesia or drilling) may have a lower incidence of fear, which could explain this phenomenon.

In the present study, despite the significant associations between many factors and dental fear, approaching the dentist’s office was a leading predictor. Approaching the dentist’s office can trigger negative thoughts and beliefs about dental procedures and associated discomfort or anxiety, leading to dental fear, even in individuals who have not previously had negative experiences with dental procedures.

However, it is important to note that various etiological factors may also contribute to the development of dental fear and anxiety, and the exact cause may vary from person to person [49]. For instance, an individual who exhibits anxiety traits and has had a bad experience may be more likely to experience dental fear when approaching the dentist’s office. Furthermore, an interaction of all the factors cannot be overlooked, and the concept that Liddell and Locker summarised in their statement, “It is impossible to say from this study whether the experiences were, in fact, very traumatic, or whether the subjects were more sensitive to them,“ applies to the role of approaching the dentist’s office as a leading predictor of dental fear as well [50]. In other words, while approaching the dentist’s office may be a leading predictor of dental fear, other factors such as past experiences, beliefs, seeing the dentist, needles and drills may also play a role in developing dental fear.

The findings from the qualitative study highlight key findings on dental fear and anxiety, categorized under four major themes: physical reactions, perceptions and fears of procedures, gender and environmental factors, and interactions with dentists as shown in Fig. 1. Based on the responses of patients, this study finds that dental procedures often trigger acute stress reactions like increased heart and breathing rates, aligning with existing literature. Patients often experience existential fears and find dental procedures unpleasant, resonating with research on “catastrophic thinking” and the sensory aspects of dental fear. The study highlights gender-related admission of fear; women are more likely to admit fear, while men underreport it due to societal norms. The environment, such as the dental office itself, can also trigger anxiety. Patients often feel that dentists lack empathy and feel vulnerable in the dental chair, which aligns with research emphasizing the importance of patient-dentist interactions in dental fear. The findings reinforce and expand upon existing research, suggesting the need for multi-dimensional approaches in future studies and interventions for dental fear and anxiety.

The quantitative and qualitative results of the study complement each other in filling a significant gap in the literature on dental fear in Pakistan. The quantitative analysis, based on 273 participants, provides robust statistical data on the prevalence of dental fear, particularly noting higher scores among females and those undergoing oral surgical treatment. It also validates the Urdu translation of Kleinknecht’s Dental Fear Survey (DFS) as a reliable tool for this specific population, which had not been previously studied. On the other hand, the qualitative component, featuring in-depth interviews with 25 patients, adds nuanced insights into the lived experiences and perceptions of those with moderate to high dental fear. This qualitative data elucidates the elements contributing to dental fear scores, enriching the statistical findings with contextual understanding. Together, these two methods offer a comprehensive view of dental fear, enabling more effective strategies for dental care in the region.

The combined qualitative and quantitative results of this study serve to both strengthen and expand our understanding of dental fear and anxiety in our population. By integrating the findings with existing literature, the study has enriched the academic discourse on dental fear and provided healthcare practitioners with insights that could potentially inform more empathetic and effective treatment methods.

### Recommendations

The purpose of addressing dental fear issues is to improve oral health and increase the use of dental care services. In this mixed methods study, we have suggested specific strategies for preventing the amplification of fears to prevent barriers to future care.

Establishing dental fear clinics would also help alleviate patients’ dental fear. The first visit to these clinics should always be preventative, emphasising oral health promotion. A fearful patient can receive emergency treatment or preventive care in these clinics, making subsequent dental treatments less painful and decreasing their anxiety and fear [2]. The model of dental fear clinics can be adopted from Netherland, where highly anxious dental patients are treated with the aid of behavioural management techniques, intravenous sedative agents and general anaesthesia. The primary goal, besides lowering anxiety, is to deliver needed restorative treatment [51].

Dentists can be instructed to pay close attention to patients who refuse to admit their fear of dentistry. Fear among patients can be reduced by having a calm and relaxing conversation between the dentist and the patient prior to treatment [30]. The conversation with the patient predicts their apprehension capacity and probable psychological response to ongoing treatment [52].

Dentists should also use less embarrassing language to encourage people to express and deal with dental fears [27]. By doing so, they will better understand the concerns of their patients, educate them, and reduce fear of specific procedures. For example, “Your teeth are in terrible shape. Have you been neglecting your oral hygiene?” is an embarrassing language. On the other hand, using language such as “It looks like there are some areas we can work on. Let’s discuss a treatment plan” is less embarrassing. Another example of embarrassing language is ““Your dental hygiene is terrible. You need to take better care of your teeth”. However, same statement can be said in this manner “Oral hygiene plays an important role. Let’s work on improving your routine”.

Furthermore, if a dentist knows that a patient fears needles or drill, strategies such as providing music or removing or covering needles from sight can help reduce patient fear. A second strategy involves opening sterile dental instruments only when the patient is seated [47]. The purpose of this practice is to ensure that patients are reassured of the cleanliness of the equipment and that there is no risk of infectious diseases before they are seated for their procedure.

Through the above techniques, the dentist can sensitively address dental fear during dental treatment and make patients more willing to seek dental care. Therefore, dentists must receive enhanced training to address patient fears and provide pain management and patient-centred dental care [47].

Some limitations should be discussed. In any questionnaire survey, respondents might hide their true feelings or underestimate their fears, anxieties, and unpleasant feelings regarding dental care. However, to overcome this issue, patients were asked to fill in responses while seated in the dental chair before starting the clinical examination. The patients were also allowed to communicate in detail about their dental fears during in-depth interviews. It is essential to remember that this study comprises a small sample of the urban population of a single teaching hospital. It is impossible to generalise the results based on such a small survey. However, the results of this study can be applied to other teaching hospitals in Pakistan that provide dental care to the urban population. In this study, the Urdu translations of DFS showed acceptable internal consistency and construct validity. Thus, these measures appear to operate similarly in Urdu and other languages. For research purposes on Pakistani populations, the DFS may be preferred due to its increased comprehensiveness. Moreover, fearful patients might appreciate the DFS since it includes items describing specific stimuli, they find fear-provoking [2]. In addition, the study evaluates dental fear among patients receiving different dental treatments. Additionally, the qualitative aspect of this study, involving interviews with individuals exhibiting moderate to high dental fear, offers a richer understanding of the lived experiences associated with dental fear. Further qualitative studies can delve deeper into personal narratives, uncovering unique cultural or societal factors contributing to dental fear in the region.

## Conclusion

The Urdu translation of Kleinknecht’s Dental Fear Survey is a reliable and valid instrument for assessing dental fear in Pakistan based on the findings of this study. This tool promises to aid dental professionals in understanding and addressing dental fears more effectively, ultimately improving dental care quality and patient experiences in the region.

Patients perceive surgical and restorative procedures as unpleasant and threatening; therefore, increasing signs of fear may be observed. It was noted that “the heart beats faster” and “the breathing rate increases” were the top two physiological responses. Female were more likely to admit to having strong fears than their male counterparts. Effective pain management strategies, distraction techniques, and behavioural interventions can play a pivotal role in reducing dental fear and enhancing patients’ overall experience.

## Data Availability

The data that support the findings of this study are available from the corresponding author upon reasonable request.
